# Tandem isomerization/telomerization of long chain dienes

**DOI:** 10.3389/fchem.2014.00037

**Published:** 2014-06-13

**Authors:** Laura Torrente-Murciano, David J. Nielsen, Kingsley J. Cavell, Alexei A. Lapkin

**Affiliations:** ^1^Department of Chemical Engineering, University of BathBath, UK; ^2^Department of Chemistry, Cardiff UniversityCardiff, UK; ^3^Department of Chemical Engineering and Biotechnology, University of CambridgeCambridge, UK

**Keywords:** tandem reaction, transition metals, carbenes, C-C coupling reactions, isomerization, telomerization

## Abstract

The first example of a tandem reaction involving double-bond migration in combination with telomerization is reported. Homogeneous and heterogeneous Ru catalysts were employed as isomerization catalysts, and telomerization was realized using a homogeneous Pd(0) precursor complex with a N-heterocyclic carbene (IMes) ligand. Overall conversions approaching 60% were achieved with the best selectivity to telomerization products of 91% attained at 11% conversion. Conversion was markedly higher in the presence of longer-chain alcohol (1-butanol) as the nucleophile (telogen).

## Introduction

Telomerization reactions, defined as the dimerization of dienes with incorporation of a nucleophile, have a major synthetic potential (Smutny, 1967; Zapf and Beller, 2002). The versatile range of nucleophiles used in this reaction, from alcohols (Krotz et al., 2001; Jackstell et al., 2002; Palkovits et al., 2008), amines (Maddock and Finn, 2000; Grotevendt et al., 2007) and carbon dioxide (Behr et al., 2007), indicates its synthetic flexibility in a variety of applications. However, until recently telomerization studies have been limited to C4-C5 dienes (butadiene and isoprene) due to their high reactivity (Benvenuti et al., 1999; Jackstell et al., 2007), limiting the range of feedstocks that can be used in the reaction. Following advances in the understanding of the bis-allyl monometallic mechanism (Behr and Urschey, 2003) including parallel catalytic cycles for the formation of lineal and branched telomerization products and dimerization by-products (Vollmuller et al., 2000; Jackstell et al., 2004) and the consequent catalysis development with the use of nucleophilic carbine (NHC) based catalysts (Clement et al., 2008), we have recently reported the telomerization of long-chain dienes (1,3-pentadiene and 1,3-hexadiene) with primary alcohols using homogeneous Pd catalysts with NHC ligands (Torrente-Murciano et al., 2010a) adding to another previous example of long-chain dienes telomerization of C_10_ terpene myrcene (Behr et al., 2010; Lopes et al., 2011).

**Figure 1:**
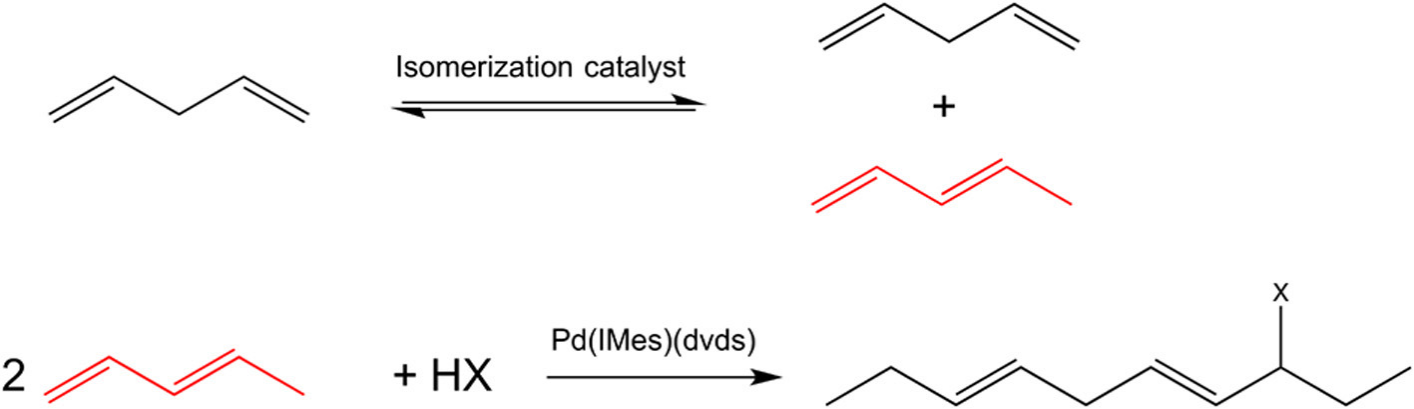
**A scheme of a tandem isomerization—telomerization of long-chain dienes for the case of pentadienes; the isomer selectively participating in the telomerization reaction is shown in red**.

To further extend the range of dienes used in telomerization beyond terminal conjugated dienes, an isomerization of internal and/or non-conjugated dienes could potentially be used. However, terminal conjugated dienes are thermodynamically unfavorable, with their equilibrium concentration typically below 5%. A tandem transformation involving an isomerization step followed by telomerization will overcome this thermodynamic limitation. Here, we demonstrate for the first time the feasibility of a tandem reaction of isomerization of internal linear to 1,3-conjugated dienes, followed by telomerization, leading to formation of functionalized long chain molecules, Figure 1. Both reactions are characterized by 100% atom economy [at full conversion, according to the definition (Trost, 1991)], and can be performed in environmentally acceptable solvents or solventless at mild conditions.

## Materials and methods

### Catalyst preparation

Homogeneous telomerization complex (1,3-dimesitylimidazol-2-ylidene)-palladium(0)-η^2^,η^2^-1,1,3,3-tetramethyl-1,3-divinyl-disiloxane (Pd(IMes)(dvds)) was synthesized by reacting the palladium (0) diallylether complex [Pd_2_(dae)_3_] (dae = diallylether) with 1,3-dimesitylimidazolin-2-ylidine carbene (IMes) in 1,1,3,3-tetramethyl-1,3-divinyl-disiloxane (dvds) at −30°C in THF. Suitable crystals for X-ray crystallography were obtained by crystallization from pentane at low temperatures (<0°C) (Jackstell et al., 2004).

Heterogeneous isomerization Ru catalysts were prepared by wet impregnation of the support with a solution of RuCl_3_ for 2 h. After this time, the catalyst was washed and dried at 80°C under vacuum prior its reduction with an aqueous solution of NaBH_4_ (0.1 M) at room temperature. Titanate nanotubes (Ti-NT) were synthesized following a hydrothermal method described elsewhere (Torrente-Murciano et al., 2010b) and were used as catalytic support without any further treatment.

### Catalytic tests

All reactants were purchased from Fisher Chemicals and used without further purification. Reactions were carried out in 15 mL Ace Pressure Tubes equipped with an FETFE® O-ring which sits under the top rim of a PTFE bushing. The heavy-wall borosilicate glass tubes are rated for 11 bar. The reaction temperature (in the range of 70–130°C) was controlled with an oil bath and a temperature controller. Specific reaction temperatures are given in the summary Tables 1–3. In a typical experiment the desired quantity of the catalysts (specific quantities are given in Table footnotes), solvent and reactant were introduced into the tube together with a stirrer bar; nitrogen was bubbled to remove air. An initial sample (time = 0) was taken for analysis. The tube was then sealed and introduced into the oil bath to start the reaction. After a certain reaction time the tube reactor was placed into an ice-bath to quench the reaction and condense the volatiles. Samples were analyzed in duplicate by gas chromatography using a Varian GC CP-3900 instrument, equipped with a non-polar capillary column AT-5 (5% phenyl/95% methylpolysiloxane, 30 m × 0.32 mm), an autosampler and a Flame Ionization Detector (FID). 1 mL min^−1^ He flow was used as a carrier gas; the injector and detector temperatures were 250 and 315°C respectively. The oven temperature programmes were: (1) 40°C for 5 min, ramp to 80°C at 5° min^−1^, ramp to 200°C at 15° min^−1^ for isomerization reaction and (2) 40°C for 5 min, ramp to 100°C at 10° min^−1^, ramp to 180°C at 8° min^−1^, ramp to 220°C at 20° min^−1^ for the telomerization products. External calibration was carried out to cover the range of concentrations of the analyzed components using analytical standards with a purity >99% and decane as internal standard. The material balance was closed in all reactions to within ±5% and the accuracy of determination of concentrations was better than ±5% based on five consecutive measurements of reactants standards of known concentrations.

**Table 1:** **Tandem isomerization—telomerization of 1,4-pentadiene catalyzed by different isomerization catalysts and Pd(IMes)(dvds) as the telomerization catalyst**.

**Isomerization Catalyst**	**Nucleophile/ solvent**	**T/°C**	**Isomerization conversion/%**	**Telomerization conversion in tandem reaction/%**	**Telomerization selectivity/%**
–	MeOH	130	–	–	–
RuCl_3_	MeOH	130	95.8	11.3	91.3
RuCl^a^_3_	MeOH	130	89.8	9.9	91.2
RuCl_3_	1-butanol	110	58.7	9.3	27.2
RuCl_3_	1-butanol	130	98.0	41.4	19.2
RuHCl(PPh_3_)_3_	1-butanol	110	98.0	28.3	34.9
RuHCl(PPh_3_)_3_	1-butanol	130	99.0	59.1	27.2
10.1% Pd^2+^/Ti-NT	MeOH	130	68.3	0.5	51.7
6.3% Rh^3+^/Ti-NT	MeOH	130	85.3	1.5	71.3
4.9% Ru^3+^/Ti-NT	MeOH	130	96.9	2.4	71.4
4.9% Ru^3+^/Ti-NT^a^	MeOH	130	88.9	4.1	83
4.9% Ru^3+^/Ti-NT	1-butanol	110	25.6	–	–
4.9% Ru^3+^/Ti-NT	1-butanol	130	57.9	3.9	100
5%Ru/C	1-butanol	110	93.5	2.3	16.6
5%Ru/C	1-butanol	130	99.0	3.0	18.2

Reaction conditions: 1,4-pentadiene (1.3 M), solvent: 5 mL dry 1% NaOMe MeOH (or 1-butanol), 1 mL decane as internal standard. Time: 19 h. Isomerization catalyst: 2.5·10^−5^ moles for homogeneous and 0.2 g for heterogeneous. Telomerization catalyst: 8·10^−6^ moles of Pd(IMes)(dvds) + 4 eq. (IMes) ligand.

a Addition of 1,3-pentadiene (0.7 M).

**Table 2:** **The effect of reaction temperature in the telomerization of 1,3-pentadiene with methanol catalyzed by Pd(IMes)(dvds)**.

**T/°C**	**TON^a^**	**Conversion/%**	**Selectivity/%**
70	157	8.4	97.9
90	405	21.7	98.8
110	352	18.8	93.7
130	226	12.1	91.6

Reaction conditions: 1,3-pentadiene (2 M). Solvent: 5 mL dry 1%NaOMe MeOH, 1 mL decane as internal standard. Time: 7 h. Catalyst: 8·10^−6^ moles of Pd(IMes)(dvds) + 4 eq. (IMes) ligand.

a TON calculated as number of moles of 1,3-pentadiene converted per mole of Pd(IMes)(dvds).

**Table 3:** **Effect of the presence of base NaOMe on the isomerization of 1,5-hexadiene**.

**Catalyst**	**T/°C**	**Presence of base**	**Conversion @ 0.5 h/%**	**Conversion @ 3 h/%**
RuHCl(PPh_3_)_3_	110	No	35.7	52.8
RuHCl(PPh_3_)_3_	110	Yes	2.5	4.4
4.9% Ru^3+^/Ti-NT	130	No	8.7	42.5
4.9% Ru^3+^/Ti-NT	130	Yes	1.2	6.6
5%Ru/C	130	No	17.5	40.1
5%Ru/C	130	Yes	5	11.1

Reaction conditions: 1,5-hexadiene (2 M). Solvent: 5 mL dry methanol, 1 mL decane as internal standard. Time: 3 h. Catalyst: 2.5·10^−5^ moles for homogeneous and 0.2 g for heterogeneous. Base: 1% NaOMe.

## Results

### Tandem isomerization/telomerization of 1,4-pentadiene

The first results for the conversion of a non-conjugated diene, 1,4-pentadiene, into telomerization products were obtained by a one-pot combination of the isomerization reaction in which 1,4-pentadiene was converted into its terminal conjugated isomer 1,3-pentadiene, followed by its telomerization with methanol or 1-butanol. This work has been inspired in our previous feasibility study of the telomerization of long chain 1,3-conjugated dienes (C_5_ and C_6_) using homogeneous palladium-based carbene catalysts (Torrente-Murciano et al., 2010a).

Table 1 shows the isomerization and telomerization conversion results of this tandem system using a variety of homogeneous and heterogeneous ruthenium and palladium based isomerization catalysts in combination with Pd(IMes)(dvds) [(1,3-dimesitylimidazolin-2-ylidene)-Pd-(dvds), dvds=1,1,3,3-tetramethyl-1,3-divinyl-disiloxane] as telomerization catalyst under different reaction conditions.

Conversion values were calculated based on the disappearance of 1,4-pentadiene. The telomerization selectivity is the percentage of telomerization products vs. by-products such as dimers and trimers of the dienes. Telomer isomer distribution was not determined at this stage as the main focus of this work is to determine the feasibility of the tandem reaction and thus, the overall conversion to telomerization products.

The absence of an isomerization catalyst resulted in a negligible overall conversion with no presence of the telomere products. Several catalytic systems were used for the isomerization step, from homogeneous Ru catalysts to supported Rh, Ru, and Pd metallic and ionic catalysts. Building on our previous results that had shown the effectiveness of titanate nanotubes as a nanostructured support for heterogenization of Pd species in the double bond migration reaction (Torrente-Murciano et al., 2007), their use has been further explored for rhodium and ruthenium ionic species. Ru^3+^/Ti-NT catalysts have shown a higher isomerization conversion in the tandem system compared to its palladium and rhodium counterparts even at a lower metal content.

The combination of homogeneous Ru isomerization catalysts with the Pd carbene catalyst achieved good conversion levels of non-conjugated dienes to the target telomerization products. Specifically, when RuCl_3_ was used as an isomerization catalyst, the overall tandem conversion value attained was 11.3%. In the corresponding direct telomerization of 1,3-pentadiene under the same reaction conditions, the achieved conversion was 29%. Clearly, higher conversions in telomerization of the conjugated dienes could be obtained with this telomerization catalyst under the optimized reaction conditions (Torrente-Murciano et al., 2010a).

Generally, the heterogeneous isomerization catalysts used in this study show a lower activity than their homogeneous counterparts, except in the case of Ru on carbon (5% Ru/C) where similar conversions were obtained. Nevertheless, in all these cases, the use of a heterogeneous isomerization catalyst dramatically decreases the telomerization conversion.

### Individual effects of the tandem reaction conditions

In this work, results for one telomerization catalyst, Pd(IMes)(dvds) are shown, although more were screened: homogeneous Pd catalysts with PPh_3_ ligands show high activity toward the telomerization of 1,3-pentadiene, but their activity drops at temperatures above 70°C due to formation of palladium black (Torrente-Murciano et al., 2010a). On the other hand, Pd(0) complexes with nucleophilic carbene (NHC) ligands usually show enhanced stabilities; Pd(IMes)(dvds) is thermally stable at 90°C in the presence of methanol. Further thermal stabilization is achieved by the use of long-chain alcohols as nucleophile/solvent in the telomerization reaction, which concomitantly increases the solubility of the dienes leading to higher conversions (Torrente-Murciano et al., 2010a). Elevated temperatures were required to attain reasonable activities in the isomerization reactions. However, temperatures above 90°C have negative influence on conversion in the telomerization of the conjugated diene with this catalyst, as shown in Table 2.

Tandem reactions were carried out in the presence of 1% NaOMe base. In the absence of a base no conversion was achieved at 70°C and even at 90°C in the telomerization of 1,3-pentadiene with butanol with Pd(IMes)(dvds) catalyst.

However, an opposite effect of the presence of the base was found in the isomerization step. Table 3 shows the drop in activity for both the homogeneous and heterogeneous Ru catalysts in the isomerization of 1,5-hexadiene in the presence of NaOMe base.

## Discussion

A series of homogeneous and heterogeneous isomerization catalysts were tested in combination with the homogeneous Pd(IMes)(dvds) telomerization catalysts for the one-pot conversion of non-conjugated 1,4-pentadiene into telomerization products, as shown in Table 1. Amongst the studied catalysts and reaction conditions, the combination of RuHCl(PPh_3_)_3_ and Pd(IMes)(dvds) using 1-butanol as nucleophile/solvent at 130°C presents the highest value of telomerization conversion (59.1%), although the selectivity to telomerization products with respect to dimerization is relatively low (27.2%). Considerably higher values of telomerization selectivity were obtained when methanol was used as a nucleophile/solvent with homogeneous ruthenium-based catalysts or heterogeneous isomerization catalysts supported on titanate nanotubes.

The main challenge associated to this tandem system is matching the rates of isomerization and telomerization reaction steps, while keeping catalysts stable under the reaction conditions. It is known, that in the absence of the reactant diene, the Pd NHC telomerization catalysts quickly deactivate (Torrente-Murciano et al., 2010a). Thus, high isomerization conversion *and* rate are essential to achieve good conversion in the tandem reaction to the product telomers without compromising the compatibility of the catalysts. However, relatively low rates of isomerization were achieved with the studied range of catalysts, requiring relatively high reaction temperatures (>100°C) to achieve sufficiently high isomerization conversions. However, despite the enhanced stabilities of Pd-carbene based catalysts, a decrease in conversion of Pd(IMes)(dvds) was observed at temperatures above 90°C as shown in Table 2, directly related to the catalyst stability at high temperatures. As selectivity remains reasonably high, the loss of activity is attributed to the loss of active form of the catalyst, also suggesting that dimerization side reaction was not specially favored at high temperatures.

High isomerization conversions were obtained in most of the studied cases, being the level of conversion directly associated to the nature of the isomerization catalyst. In general, higher isomerization conversion values were obtained with homogeneous ruthenium-based catalysts compared to their heterogeneous counterparts. However, in all cases, the presence of a heterogeneous catalyst presents a detrimental effect on the telomerization conversion. This decrease cannot be attributed to adsorption of the Pd NHC catalysts onto the heterogeneous support as its presence has a negligible effect on the catalyst performance in the telomerization reaction alone. In other words, the presence of solid supports has no effect on the telomerization reaction of 1,3-pentadiene under the same reaction conditions. Instead, it is believed that the observed detrimental effect is caused by a combination of the lower reaction rate offered by heterogeneous isomerization catalysts compared to homogeneous ones and the potential migration of telomerization ligands to the isomerization metal active sites as the formation of visible palladium black increased with the increase in the isomerization metal loading.

The presence of a base (NaOMe) is critical for the progress of the telomerization reaction, as it increases the concentration of MeO- anions in solution, which is essential during nucleophilic attack in the telomerization reaction (Nielsen and Cavell, 2006). However, its presence has a detrimental effect on the rate of isomerization, as shown in Table 3. This decrease in the rate of the first step of the tandem system is translated into low isomerization conversions and, consequently, low concentrations of the terminal conjugated diene in the reaction medium. As the telomerization rate is proportional to the concentration of terminal dienes, this observation explains the low yield in the telomerization step in the tandem reaction compared to that for telomerization of pure 1,3-pentadiene at the same reaction conditions. Therefore, reduction in the availability of the conjugated diene has a significant impact on the second reaction in the tandem system. Following these observations, in a number of tandem experiments, a 1:2 mixture of the terminal conjugated diene (1,3-pentadiene) and the non-conjugated one (1,4-pentadiene) was used to increase the availability of the reactant to the telomerization catalyst and thus increase its stability. However, this resulted in the reduction of the isomerization conversion due to competition of the internal and external dienes for the catalyst active species, as shown in Table 1 rows 3 and 11. Consequently, the overall isomerization conversion slightly decreases in this case, although having a negligible effect on the telomerization step.

In conclusion, we have reported for the first time a tandem isomerization-telomerization reaction, thus extending synthetic utility of the atom-efficient telomerization reaction. The observed trends with respect to the effects of temperature, base and relative rates of reactions suggest ways toward developing more effective, optimized, catalytic systems for such tandem reactions.

### Conflict of interest statement

The authors declare that the research was conducted in the absence of any commercial or financial relationships that could be construed as a potential conflict of interest.
